# Conditional testing of multiple variants associated with bone mineral density in the *FLNB* gene region suggests that they represent a single association signal

**DOI:** 10.1186/1471-2156-14-107

**Published:** 2013-10-31

**Authors:** Benjamin H Mullin, Cyril Mamotte, Richard L Prince, Tim D Spector, Frank Dudbridge, Scott G Wilson

**Affiliations:** 1 Department of Endocrinology & Diabetes, Sir Charles Gairdner Hospital, Nedlands, Western Australia, Australia; 2 School of Biomedical Sciences and CHIRI Biosciences, Curtin University of Technology, Bentley, Western Australia, Australia; 3 School of Medicine and Pharmacology, University of Western Australia, Nedlands, Western Australia, Australia; 4 Twin & Genetic Epidemiology Research Unit, St Thomas’ Hospital Campus, King’s College London, London, UK; 5 Faculty of Epidemiology and Population Health, London School of Hygiene and Tropical Medicine, London, UK

**Keywords:** Bone mineral density, *Filamin B*, SNP, Osteoporosis

## Abstract

**Background:**

Low bone mineral density (BMD) is a primary risk factor for osteoporosis and is a highly heritable trait, but appears to be influenced by many genes. Genome-wide linkage studies have highlighted the chromosomal region 3p14-p22 as a quantitative trait locus for BMD (LOD 1.1 - 3.5). The *FLNB* gene, which is thought to have a role in cytoskeletal actin dynamics, is located within this chromosomal region and presents as a strong candidate for BMD regulation. We have previously identified significant associations between four SNPs in the *FLNB* gene and BMD in women. We have also previously identified associations between five SNPs located 5*'* of the transcription start site (TSS) and in intron 1 of the *FLNB* gene and expression of *FLNB* mRNA in osteoblasts *in vitro*. The latter five SNPs were genotyped in this study to test for association with BMD parameters in a family-based population of 769 Caucasian women.

**Results:**

Using FBAT, significant associations were seen for femoral neck BMD Z-score with the SNPs rs11720285, rs11130605 and rs9809315 (*P = 0.004 – 0.043*). These three SNPs were also found to be significantly associated with total hip BMD Z-score (*P = 0.014 – 0.026*). We then combined the genotype data for these three SNPs with the four SNPs we previously identified as associated with BMD and performed a conditional analysis to determine whether they represent multiple independent associations with BMD. The results from this analysis suggested that these variants represent a single association signal.

**Conclusions:**

The SNPs identified in our studies as associated with BMD appear to be part of a single association signal between the *FLNB* gene and BMD in our data. *FLNB* is one of several genes located in 3p14-p22 that has been identified as significantly associated with BMD in Caucasian women.

## Background

Postmenopausal osteoporosis is a systemic bone disease that is characterised by low bone mass and disturbed micro architecture of bone tissue, resulting in decreased bone strength and a corresponding increase in the risk of fracture [1]. Bone mass peaks in early adult life, but declines in postmenopausal women due to a reduction in oestrogen production which has direct effects on bone as well as calcium handling by the intestine and renal system [2]. In addition to these effects and environmental factors, there is a strong genetic effect on peak bone mass, bone loss and fracture rates in postmenopausal women [3]. Twin and family studies suggest that 50-85% of the variance in peak bone mass [4-7] and 25-68% of the variance in osteoporotic fracture is heritable [8-10].

The whole genome linkage scanning approach has identified multiple quantitative trait loci (QTL) for bone mineral density (BMD) [11], strongly suggesting that genetic influence for the phenotype is mediated through multiple genes. The 3p14-p22 region of the human genome has been identified as a QTL for BMD in multiple genome-wide linkage studies (LOD 1.1 - 3.5) [12-15]. The *Filamin B* (*FLNB*) gene, which is thought to have a role in cytoskeletal actin dynamics [16], is located within this chromosomal region and presents as a strong candidate for BMD regulation. Mutations within the *FLNB* gene have been implicated in a variety of genetic disorders characterised by skeletal malformation, some of which include: spondylocarpotarsal synostosis syndrome [17-19], Larsen syndrome [18,20], atelosteogenesis types I and III [18,21] and boomerang dysplasia [22]. Associations have also been identified between polymorphism in *FLNB* and human stature variation in a genome-wide association study [23].

We have previously identified significant associations between four single nucleotide polymorphisms (SNPs) in the *FLNB* gene and BMD in Caucasian women [24]. Two of these SNPs were identified as being in moderate to strong linkage disequilibrium (LD) with five other SNPs located either 5′ of the transcription start site (TSS) or in intron 1 of the gene, all five of which were significantly associated with expression of *FLNB* mRNA in 96 human osteoblast cell lines [24]. Based on this data, we decided to perform a follow up to our previous study [24] and examine these five SNPs in relation to BMD parameters in a family-based population of Caucasian women. Genotype data for any significant variants would then be combined with the genotype data for the four *FLNB* SNPs previously associated with BMD [24] in a conditional analysis to determine whether multiple loci exist in the *FLNB* gene that are independently associated with BMD.

## Results

**Figure 1:**
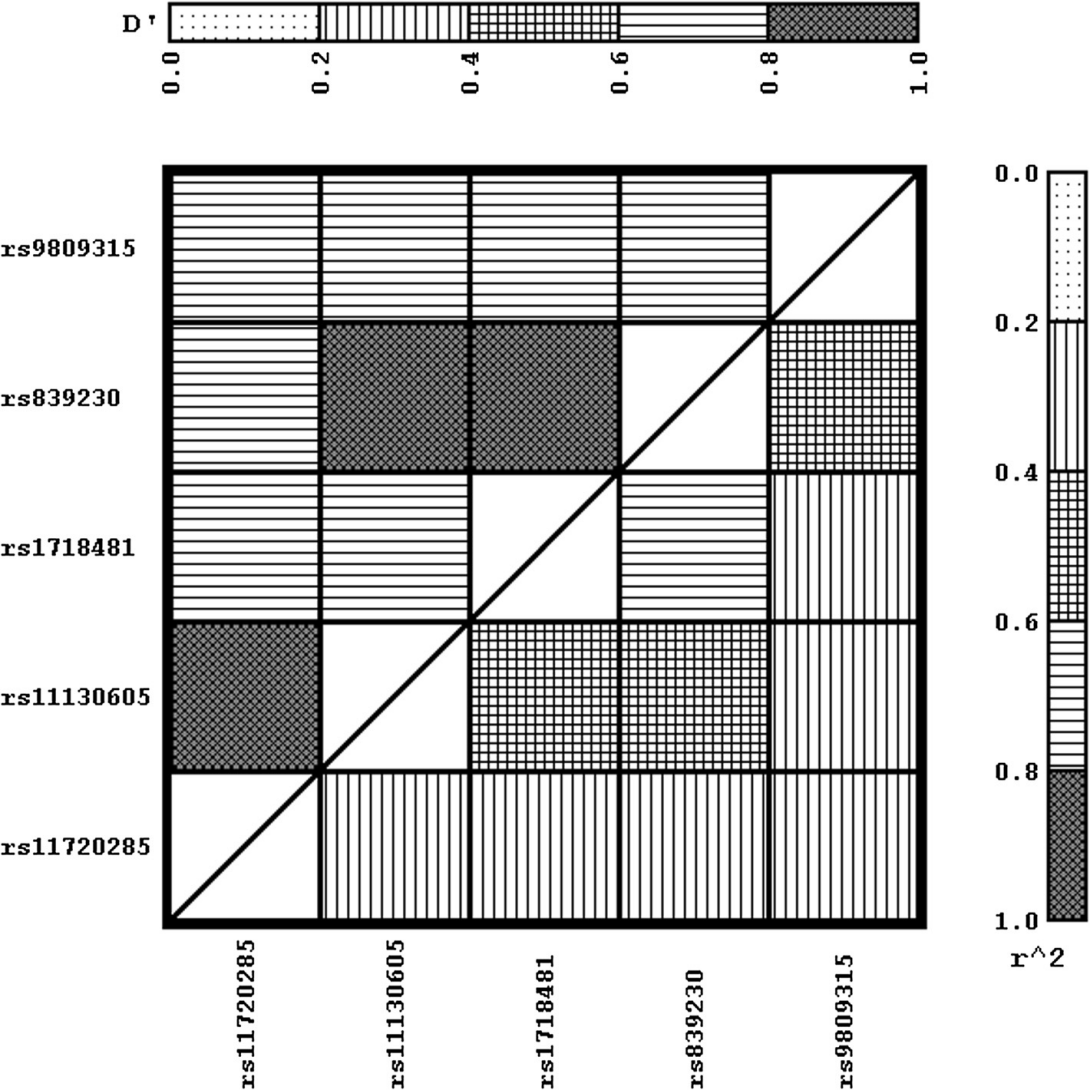
LD analysis of the 5 SNPs genotyped in this study.

The demographic and morphometric characteristics of the population are detailed in Table 1. There are a large number of osteoporotic individuals in this population, resulting in a negative mean BMD Z-score observed at each site studied. All 5 SNPs genotyped were in Hardy-Weinberg equilibrium as determined using a χ^2^ test. LD analysis revealed that none of the 5 SNPs genotyped were in LD of r^2^ > 0.8 with each other (Figure 1). An additional LD analysis revealed that one of the 5 SNPs genotyped in this study, rs839230, is in LD of r^2^ > 0.8 with rs704529 which was genotyped in our previous study [24]. However, none of the other 4 SNPs genotyped in this study were in LD of r^2^ > 0.8 with any of the other SNPs genotyped in our previous study [24]. The chromosomal position and allele distribution of the 5 SNPs genotyped in this study and the 13 SNPs genotyped in our previous study [24] is detailed in Table 2.

**Table 1:** Demographics and bone density of the population studied

**Variable**	**Population mean**
Age (years)	54.2 ± 12.7 (769)
Weight (Kg)	62.7 ± 11.3 (699)
Total hip DXA BMD (mg/cm^2^)	801 ± 136 (760)
Total hip BMD Z-score	-0.4 ± 1.0 (760)
Femoral neck DXA BMD (mg/cm^2^)	700 ± 133 (749)
Femoral neck BMD Z-score	-0.4 ± 1.1 (749)
Spine L1-L4 DXA BMD (mg/cm^2^)	855 ± 158 (767)
Spine BMD Z-score	-0.7 ± 1.3 (767)

Results are given as *mean ± SD (number of measurements).*

**Table 2:** **Position and allele distribution of all *FLNB *** **variants genotyped in this and our previous study**

**SNP**	Chromosome position*	Location*	**Major/minor allele**	**Minor allele frequency (%)**
rs11720285	57961370	5*'* of TSS	A/C	25.1
rs7637505^†^	57968393	5*'* of TSS	A/T	29.4
rs6445938^†^	57974822	5*'* of TSS	A/G	23.1
rs11130605	57989169	5*'* of TSS	C/T	39.2
rs6798382^†^	57991811	5*'* of TSS	G/A	28.6
rs4681772^†^	57992512	5*'* of TSS	A/G	32.1
rs1658351^†^	58013573	Intron 1	A/G	33.5
rs1718481	58025903	Intron 1	G/A	42.7
rs704529^†^	58036651	Intron 1	A/G	36.2
rs839230	58036792	Intron 1	G/A	36.2
rs9809315	58050265	Intron 1	C/T	30.2
rs9822918^†^	58057684	Intron 1	C/A	44.6
rs2177153^†^	58092346	Intron 11	A/G	31.8
rs1131356^†^	58109162	Exon 21, Asp > Asn	G/A	22.9
rs12632456^†^	58118555	Exon 26, Val > Met	G/A	23.2
rs2001972^†^	58123249	Intron 28	C/A	37.6
rs4284952^†^	58126223	Intron 29	C/A	34.6
rs4234386^†^	58150433	Intron 43	G/A	24.4

*Relative to GenBank reference sequence NM_001457, Genome Build 37.5.

^†^Variant genotyped in our previous study [24].

### Locus specific analyses: effects of individual SNP genotypes on phenotypic data

Using FBAT, significant associations were seen for femoral neck BMD Z-score with the SNPs rs11720285, rs11130605 and rs9809315 (*P = 0.005*, *0.043* and *0.004* respectively). These three SNPs were also found to be significantly associated with total hip BMD Z-score (*P = 0.014*, *0.026* and *0.022* respectively). No significant associations were observed between any of the 5 SNPs examined and spine BMD Z-score. After correction of the data for testing 5 SNPs across 3 anatomical sites, the significant association between rs9809315 and femoral neck BMD Z-score was maintained (*P = 0.028*). Estimates of the additive genetic effect suggest that the minor alleles at rs11720285 (*C*), rs11130605 (*T*) and rs9809315 (*T*) are associated with an increased BMD Z-score at both the total hip and femoral neck sites (Table 3).

**Table 3:** **Additive value of minor allele for *FLNB *** **variants associated with BMD Z-score in this and our previous study**

**SNP**	**BMD Z-score phenotype**	**Additive value of minor allele**	***P***
rs11720285	Femoral neck	+ 0.306 (366)	0.03
	Total hip	+ 0.274 (373)	0.053
rs7637505^†^	Femoral neck	+ 0.287 (422)	0.031
	Total hip	+ 0.262 (434)	0.033
rs11130605	Femoral neck	+ 0.241 (575)	0.045
	Total hip	+ 0.268 (592)	0.03
rs9809315	Femoral neck	+ 0.304 (435)	0.014
	Total hip	+ 0.254 (448)	0.045
rs9822918^†^	Femoral neck	+ 0.397 (650)	0.002
	Total hip	+ 0.414 (668)	0.002
rs2177153^†^	Femoral neck	+ 0.537 (459)	0.0004
	Total hip	+ 0.443 (474)	0.001
rs2001972^†^	Femoral neck	+ 0.254 (547)	0.043
	Total hip	+ 0.264 (563)	0.041

Results are given as *additive effect on the trait of the minor allele relative to the more common allele (number of alleles included in analysis)*, derived from UNPHASED v3.1.5.

^†^Variant genotyped in our previous study [24].

### Haplotype analysis

A 3-SNP haplotype analysis was carried out using rs11720285, rs11130605 and rs9809315 to determine whether haplotypes of the LD blocks tagged by each SNP would prove to be more significantly associated with femoral neck or total hip BMD Z-score than in the individual SNP analysis. Six haplotypes with a frequency greater than 4% in the population were identified. Significant associations were observed with femoral neck BMD Z-score only (Table 4), although the level of significance did not surpass that observed in the individual SNP analysis for this phenotype. The *CTT* haplotype was found to have a strong positive influence on femoral neck BMD Z-score.

**Table 4:** Haplotype analysis and additive value of each haplotype relevant to femoral neck BMD Z-score

**BMD Z-score phenotype**	**Haplotype (rs11720285, rs11130605, rs9809315)**						
	*ACC allele*	*CTT allele*	*ATC allele*	*ACT allele*	*ATT allele*	*CTC allele*	*P*
Femoral neck	0 (769)	+ 0.72 (260)	+ 0.184 (148)	+ 0.044 (83)	- 0.442 (82)	- 0.164 (76)	0.005

Results are given as *additive effect on the trait of each haplotype relative to the most common haplotype (number of alleles included in the analysis)*, derived from UNPHASED v3.1.5.

### Bioinformatics analysis

The SNP rs11130605 has been identified by the HapMap Genome Browser [25] (release #27) as being in complete LD with the SNPs rs7631741 and rs7634753 in the CEU population (Utah residents with Northern and Western European ancestry). These two SNPs were therefore included in the bioinformatics analysis.

The rs11130605 polymorphism has an F-SNP score of 0.101 as it is located in a potential regulatory region. rs7634753 has an F-SNP score of 0.242 with the more common *C* allele resulting in the loss of an STRE site and the gain of N-Myc and USF sites. The rs9809315 polymorphism also has an F-SNP score of 0.242 and is located in a potential regulatory region, with the more common *C* allele resulting in loss of HSF, Dfd, CdxA and GCN4 sites. No functional information currently exists for rs11720285 and rs7631741 in F-SNP, so these variants were analysed using HaploReg [26]. Using this resource, the more common *A* allele at rs11720285 was found to result in the loss of a Sox_9 site with the gain of Evi-1_2 and Hoxa4 sites. The more common *T* allele at rs7631741 was found to result in a gain of a HMG-IY_2 site.

### Conditional analysis

The genotype data for rs11720285, rs11130605 and rs9809315 was combined with that for the four other SNPs from the *FLNB* gene that we previously identified as associated with BMD [24] for a conditional analysis. These four SNPs are rs7637505, rs9822918, rs2177153 and rs2001972. Out of these seven SNPs, rs2177153 demonstrated the strongest associations with BMD and was therefore used as the conditioning marker. Once the data had been conditioned on rs2177153, no significant associations with femoral neck or total hip BMD Z-score were observed for any of the other six SNPs (*P = 0.32 – 0.93*).

## Discussion

We previously identified 5 SNPs from the 5′ region of the *FLNB* gene that were strongly associated with expression levels of *FLNB* mRNA [24], 3 of which have been shown here to be significantly associated with age-adjusted total hip and femoral neck BMD in Caucasian women. The minor allele at each of these 3 SNP sites was associated with an increased BMD Z-score in this study and a reduced level of *FLNB* mRNA in our previous study [24]. However, when the genotype data for these 3 SNPs was combined with that for 4 *FLNB* SNPs previously identified as associated with BMD and conditioned on the most significantly associated SNP, none of the associations remained significant. This suggests that these variants represent a single association signal between the *FLNB* gene and BMD in this study population. This is not the first time that common variation within a gene that has previously been identified as underlying a rare monogenetic form of osteoporosis and/or high bone mass has been implicated in BMD regulation, other examples including the *SOST*, *CLCN7* and *LRP5* genes [27].

Although rs2177153 demonstrated the most significant associations with BMD in the study population combined dataset [24], we cannot be sure that this is the quantitative trait nucleotide responsible for the associations seen. Of the three SNPs identified in this study as associated with BMD, rs11130605 demonstrated the most significant associations with *FLNB* mRNA expression levels in our previous study [24]. This could suggest that the SNP rs11130605, or another SNP in very strong LD with it, is the causal variant responsible for the associations seen. It is interesting that F-SNP identified rs11130605 and rs9809315 as being located in potential regulatory regions. These SNPs had F-SNP scores of 0.101 and 0.242 respectively, with the median F-SNP score for a neutral SNP thought to be around 0.176 [28]. The SNP rs7634753 also had an F-SNP score of 0.242, with the common allele predicted to cause the gain of binding sites for the transcription factors N-Myc and USF. Both of these transcription factors have been shown to have a role in bone, with N-Myc having been implicated in the development of the limb bones in mice [29,30] and USF having been shown to have a role in the process of receptor activator of nuclear factor kappa-B ligand (RANKL) induced tartrate-resistant acid phosphatase (TRAP) transcription during osteoclast differentiation [31]. Variation at rs11720285 was found to alter Hoxa4 and Sox_9 sites. Hoxa4 may have a role in vertebral development in mice [32], while mutations in the human *SOX9* gene have been found to cause campomelic dysplasia [33], a disease characterised by shortness and bowing of long tubular bones, hypoplastic scapulae and narrow iliac wings. There are two non-synonymous coding changes within the *FLNB* gene that have a minor allele frequency > 1% in Caucasians, both of which were genotyped in our previous study [24] and neither of which demonstrated significant associations with BMD parameters.

A recent study published by Li et al. [34] analysed seven SNPs from the *FLNB* gene, including the SNPs rs9822918 and rs2177153 genotyped in our previous study [24], for association with BMD parameters in a case–control population of 1,080 Chinese females, 533 of whom were postmenopausal. The authors were not able to replicate the associations that we observed between rs2177153 and BMD, which is probably due to the fact that this SNP had a minor allele frequency of only 0.02 in their population compared to a mean of 0.32 in our study. However, they did observe significant associations between rs9822918 and BMD at the total hip [34]. Although providing more support of a role for the *FLNB* gene in osteoporosis, comparisons with this study and our previous study must be done with caution due to differences in study design as well as differences in the ethnicity of the study subjects.

In addition to the evidence suggesting a role for the human *FLNB* gene in bone development [17-22], there is increasing evidence to suggest that the murine *Flnb* gene also has a role in bone. *Flnb* expression has been detected in vertebral bodies obtained from mouse embryos, and it has been suggested that the gene plays a role in vertebral segmentation, joint formation and endochondral ossification [18]. Zhou et al. [35] generated mice with a targeted disruption of the *Flnb* gene and observed impaired development of the microvasculature and skeletal systems in *Flnb*-deficient embryos, few of which reached term. Those that were born were very small and had severe skeletal malformations including scoliotic and kyphotic spines, fusion of vertebral bodies, lack of intervertebral discs and reduced hyaline matrix in the extremities, thorax and vertebrae [35]. Another study published by Lu et al. [36] found that *Flnb*-deficient mice presented with shortened distal limbs and small body size, abnormal spinal curvatures, dysmorphic facial/calvarial bones and develop fusion of the ribs and vetebrae, which appeared to be caused by a delay in chondrocyte development.

Over 70 binding partners have been identified to date for the filamin proteins [37,38]. They are thought to have a role in stabilising the actin cytoskeleton, providing a link between the actin network and the cellular membranes, and mediating interactions between actin and transmembrane receptors [16]. Filamins act to maintain the structural integrity of cells by crosslinking the actin cytoskeleton into 3D structures [16,39]. The ability of filamin proteins to bind actin at their N-terminus and form tail-to-tail homodimers at their C-terminus allows them to create the orthogonal actin networks and bundles that result in gelation [40]. It has also been proposed that filamins are important during foetal development, regulating the communication between extracellular signals and the cytoskeleton to guide migration of cells into the correct anatomical sites [16].

*FLNB* is the third gene from the 3p14-p22 region of the human genome that we have found to be associated with BMD in Caucasian women, the other two being *ARHGEF3*[41] and *RHOA*[42]. Interestingly, all three of these genes appear to have a role in cytoskeletal dynamics and actin polymerisation [16,43,44]. It is possible that more than one gene from this chromosomal region may be responsible for the linkage observed between 3p14-p22 and BMD. A recently published meta-analysis of 17 genome-wide association studies identified 56 loci associated with BMD, including the *CTNNB1* gene located in 3p22.1, and 14 loci associated with risk of fracture at the genome-wide significance level [27]. Although providing some weak evidence of association for SNPs in *FLNB* with BMD, the SNPs analysed here were not identified in this meta-analysis as associated with BMD at the genome-wide significance level (*P < 5 × 10*^*-8*^). The results of the meta-analysis must be treated with caution though as allele risk modelling suggested that only 5.8% of the total genetic variance in femoral neck BMD was accounted for by the genome-wide-significant SNPs reported in the study [27]. The role of rare or private variants, functional SNPs or small insertions and deletions that are not well represented by the genome-wide association study SNP arrays due to weak LD, may explain the inability of the meta-analysis to characterise most of the source of the genetic variance.

## Conclusions

Polymorphism at rs11720285, rs11130605 and rs9809315, all of which are located either 5′ of the TSS or in intron 1 of the *FLNB* gene and have been previously reported to be associated with *FLNB* mRNA expression in osteoblasts *in vitro*[24], has been identified as significantly associated with BMD in Caucasian women in this study. The fact that these SNPs are located in non-coding regions of the gene and influence mRNA levels indicates that the associations observed are due to regulatory effects on the gene. A conditional analysis of the genotype data for these SNPs combined with those showing associations with BMD from our previous study [24] suggests a single association signal between these variants and BMD.

## Methods

### Subjects

A total of 769 women from 335 families were recruited in Australia and the UK. This family-based population included siblings recruited in 1998 for a study of the genetics of osteoporosis [13] and is the same as the family-based cohort used in our previous *FLNB* study [24] with the inclusion of one additional sibling pair. Exclusion criteria were applied where possible and included the presence of bone cancer, hyperparathyroidism, unstable thyroid disease, long term steroid use (> 5 mg/day for more than 6 months and presently on therapy), chronic immobility, rheumatoid arthritis, anorexia nervosa, osteomalacia, amenorrhea for > 6 months, premature cessation of regular menstruation or surgical oophorectomy +/- HRT (age < 40 yrs), and epilepsy with use of anticonvulsant medication for > 1 year. All subjects from the study provided written informed consent and the experimental protocols were approved by the Sir Charles Gairdner Group Human Research Ethics Committee and the St Thomas’ Hospital Research Ethics Committee.

At a clinic visit data including age, height, weight, medical, gynaecological, and lifestyle data were recorded and a blood sample collected. Dual energy X-ray absorptiometry (DXA) BMD was assessed (Hologic Inc., Bedford, MA, USA) at the lumbar spine L1-L4 and the total hip that includes an area from the femoral neck to just below the lesser trochanter. Within this area the femoral neck sub-region is widely used in clinical practice for prediction of fracture propensity and was therefore also included in the study. Due to the range of ages in this cohort, BMD data was adjusted for age prior to analysis by conversion to BMD Z-scores.

### Genotyping

Genomic DNA was extracted and purified from EDTA whole blood obtained from each subject [45]. Genotyping was performed on genomic DNA using the TaqMan assay, which utilises fluorogenic 5′ nuclease chemistry, in 384-well PCR plate format. Using this technique the genotype call rate was 99.3% and the estimated error rate was < 0.1%.

### SNP selection

5 SNPs located either 5′ of the TSS or in intron 1 of the *FLNB* gene were selected for genotyping. These were selected based on previously published data suggesting that all five SNPs are significantly associated with expression of *FLNB* mRNA in 96 human osteoblast cell cultures [24].

### Statistical analysis

Data from the cohort was analysed using the FBAT (Family Based Association Tests) software to test for association within sib-pairs, a method based on the allelic transmission disequilibrium test [46]. We used a within-family additive genetic association model and the empirical variance estimator was used to allow for prior linkage to the region. Correction for multiple testing was performed by randomly permuting phenotypes within sibships and repeating all FBAT tests on the permuted datasets. The minimum P-values were recorded for 10,000 random reassignments of the data using an automated script written in Perl. This approach was used to correct for tests of multiple SNPs within each phenotype, and also for tests of multiple SNPs across multiple phenotypes.

UNPHASED v3.1.5 was used to estimate the genetic effect size in this cohort by generating an additive value as an estimate to how each allele influences the trait value relative to the most common allele [47]. This program was also used to perform a haplotype analysis and a conditional analysis. Throughout, two-tailed P-values are reported, with *P < 0.05* considered significant. LD between the different SNPs was evaluated using the JLIN software [48].

### Bioinformatics analysis

An *in silico* analysis of SNP targets was performed using the Functional Single Nucleotide Polymorphism (F-SNP) database [49], which integrates data from 16 bioinformatics resources to help identify SNPs that may disrupt functional genomics regions. Any variants not present in the F-SNP database were analysed using HaploReg [26], a web-based tool for exploring annotations of the non-coding genome.

## Competing interests

The authors declare they have no competing interests.

## Authors’ contributions

BHM performed the genotyping, statistical analysis and drafted the manuscript. CM and RLP participated in the design and co-ordination of the study. TDS and SGW were involved in the recruitment of the study subjects and participated in study design and co-ordination. FD assisted in the statistical analysis of the data and study design. All authors read and approved the final manuscript.
